# Supplementary material for: Vortex fluidics-mediated DNA rescue from formalin-fixed museum specimens
Source: PLoS One. 2020 Jan 30;15(1):e0225807. doi: 10.1371/journal.pone.0225807 (PMC6992170; doi:10.1371/journal.pone.0225807)
Supplement: S1 Fig — (A) qPCR was used to quantify DNA encoding ATP synthase (579 bp) recovered from formalin-fixed, frozen and ground lobster tissue by VFD-processing (1 h, RT) and non-VFD (–) methods (1 h, 37 °C) at the indicated rotational speeds. DNA isolated from fresh lobster tissue provided the positive control. (B) Following qPCR, the resultant DNA was visualized by 1% agarose gel electrophoresis, which highlights the formation of primer dimers in the negative controls, including for the negative control with omitted template (no template control, NTC). Error bars indicate standard deviation (technical replicate, n = 3). (PDF) [file pone.0225807.s001.pdf]

Supplementary information

Part 1. VFD rotational speed effects on qPCR and sequencing (Fig 2)

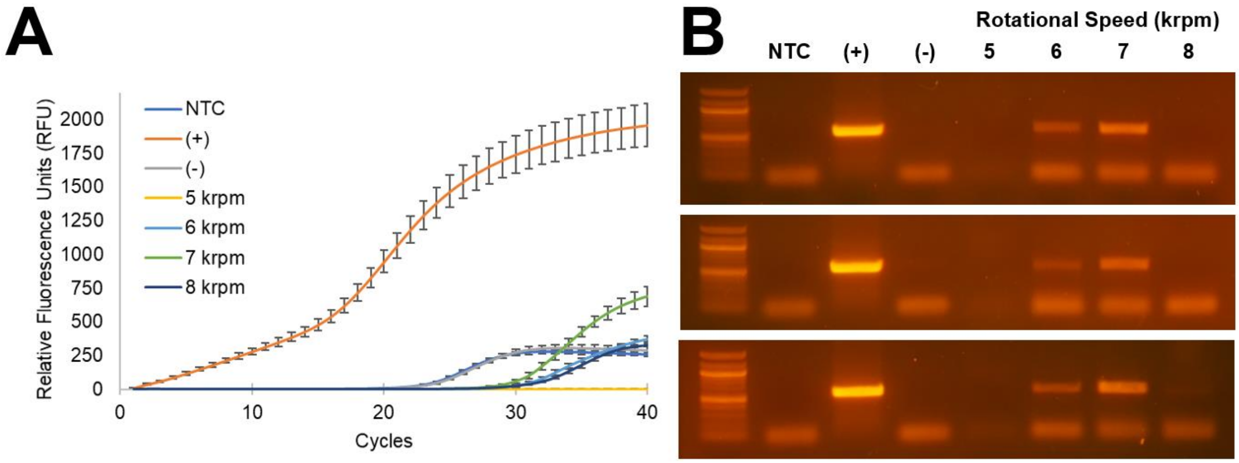

S1 Fig. Expanded versions of Fig 2B. **(A)** qPCR was used to quantify DNA encoding ATP synthase (579 bp) recovered from formalin-fixed, frozen and ground lobster tissue by VFD-processing (1 h, RT) and non-VFD (–) methods (1 h, 37 °C) at the indicated rotational speeds. DNA isolated from fresh lobster tissue provided the positive control. **(B)** Following qPCR, the resultant DNA was visualized by 1% agarose gel electrophoresis, which highlights the formation of primer dimers in the negative controls, including for the negative control with omitted template (no template control, NTC). Error bars indicate standard deviation (technical replicate, n = 3).
