# Supplementary material for: Vortex fluidics-mediated DNA rescue from formalin-fixed museum specimens
Source: PLoS One. 2020 Jan 30;15(1):e0225807. doi: 10.1371/journal.pone.0225807 (PMC6992170; doi:10.1371/journal.pone.0225807)
Supplement: S2 Fig — Sanger DNA sequencing of the target mitochondrial ATP synthase sequence from ground and sonicated then VFD-processed (9 krpm, 1 h, RT), formalin-fixed lobster tissue. Within the range of Sanger sequencing accuracy, two mutations (G2728A & G3136C, GenBank No. HQ402925) were observed as indicated. The reference sequence has been described previously [1]. (PDF) [file pone.0225807.s002.pdf]

```
Previous Sequence:  ATTCCCTACCTTTATTGAGCAGCACCCTCACGTTGAGCATTAGTTTGATCCAAAATTATGCAACTTCTTCACAATGAATT
Forward:            -------------------------------GTTGAGCATTAGTTTGATCCAAAATTATACAACTTCTTCACAATGAATT
Reverse:            ATTCCCTACCTTTATTGAGCAGCACCCTCACGTTGAGCATTAGTTTGATCCAAAATTATACAACTTCTTCACAATGAATT

TCAAGCAATTTTAGGACAAGCCACTAAAGGTTCAACTATTTTATTCATTTCTTTATTTAGATTTATTATATTCAATAACACTCTCGGGCTGCTCCCATAT
TCAAGCAATTTTAGGACAAGCCACTAAAGGTTCAACTATTTTATTCATTTCTTTATTTAGATTTATTATATTCAATAACACTCTCGGGCTGCTCCCATAT
TCAAGCAATTTTAGGACAAGCCACTAAAGGTTCAACTATTTTATTCATTTCTTTATTTAGATTTATTATATTCAATAACACTCTCGGGCTGCTCCCATAT

GTGTTTACTAGATCAAGACACCTTGCGATAACTCTTACGCTTGCTTTACCTTTATGGCTAACTTTTATTTTATTTGGTTGAATTAACCATACCCAACACA
GTGTTTACTAGATCAAGACACCTTGCGATAACTCTTACGCTTGCTTTACCTTTATGGCTAACTTTTATTTTATTTGGTTGAATTAACCATACCCAACACA
GTGTTTACTAGATCAAGACACCTTGCGATAACTCTTACGCTTGCTTTACCTTTATGGCTAACTTTTATTTTATTTGGTTGAATTAACCATACCCAACACA

TGCTTGCTCATATGGTTCCCCAAGGAACACCAGGGCTTCTGATACCATTTATAGTGTTAGTAGAAACTTTAAGAAATATTATTCGACCAGGAACTTTAGC
TGCTTGCTCATATGGTTCCCCAAGGAACACCAGGGCTTCTGATACCATTTATAGTGTTAGTAGAAACTTTAAGAAATATTATTCGACCAGGAACTTTAGC
TGCTTGCTCATATGGTTCCCCAAGGAACACCAGGGCTTCTGATACCATTTATAGTGTTAGTAGAAACTTTAAGAAATATTATTCGACCAGGAACTTTAGC

CGTTCGACTAGCAGCTAACATAATTGCAGGACATCTTTTGTTGACACTTTTAGGCAATATAGGCCCTTCTTTGTCTTTGACTTTAGTGTCGTTTTTAATA
CGTTCGACTAGCAGCTAACATAATTGCAGGACATCTTTTGTTGACACTTTTAGGCAATATAGGCCCTTCTTTGTCTTTGACTTTAGTCTCGTTTTTAATA
CGTTCGACTAGCAGCTAACATAATTGCAGGACATCTTTTGTTGACACTTTTAGGCAATATAGGCCCTTCTTTGTCTTTGACTTTAGTCTCGTTTTTAATA

CTAGCTCAAATCCTTCTTTTAATACTAGAATCTGCTGTTGCAATAATTCAATCGTATGTATTTGCTGTTCTAAGGACTTTATATGCCA
CTAGCTCAAATCCTTCTTTTAATACTAGAATCTGCTGTTGCAATAATTCAATCGTATGTATTTGCTGTTCTAAGGACTTTATATGCCA
CTAGCTCAAATCCTTCTTTTAATACTAGAATCTGCTGT-GCAAT--------------------------------------------
```

S2 Fig. Expanded version of Fig 2C. Sanger DNA sequencing of the target mitochondrial ATP synthase sequence from ground and sonicated then VFD-processed (9 krpm, 1 h, RT), formalin-fixed lobster tissue. Within the range of Sanger sequencing accuracy, two mutations (G2728A & G3136C, GenBank No. HQ402925) were observed as indicated. The reference sequence has been described previously [1].
