# Supplementary material for: Vortex fluidics-mediated DNA rescue from formalin-fixed museum specimens
Source: PLoS One. 2020 Jan 30;15(1):e0225807. doi: 10.1371/journal.pone.0225807 (PMC6992170; doi:10.1371/journal.pone.0225807)
Supplement: S3 Fig — (PDF) [file pone.0225807.s003.pdf]

# Part 2. DNA quantification by UV-Vis absorbance

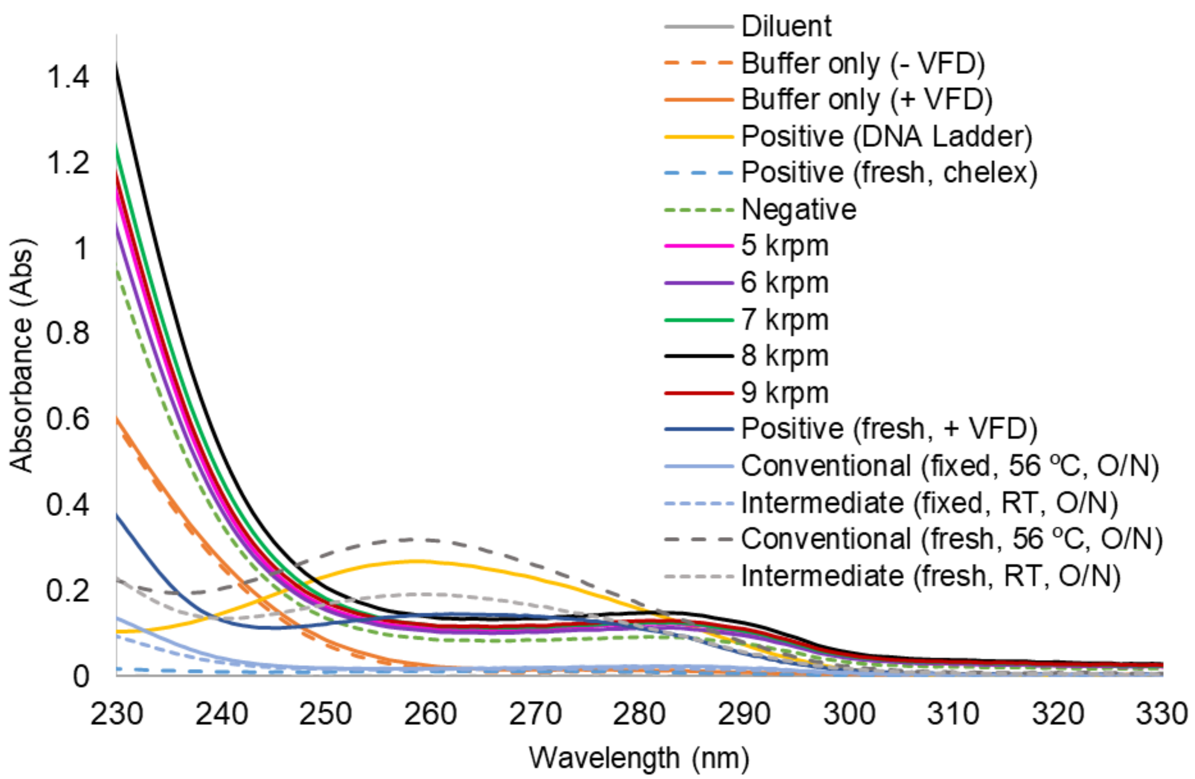


**S3 Fig.** The absorbance spectra of reaction supernatants for the fixed lobster samples and positive and negative controls given in Fig 3.
