# Supplementary material for: Vortex fluidics-mediated DNA rescue from formalin-fixed museum specimens
Source: PLoS One. 2020 Jan 30;15(1):e0225807. doi: 10.1371/journal.pone.0225807 (PMC6992170; doi:10.1371/journal.pone.0225807)
Supplement: S4 Fig — (PDF) [file pone.0225807.s004.pdf]

assay.

## Part 3. DNA quantification by SYBR Green I fluorescence

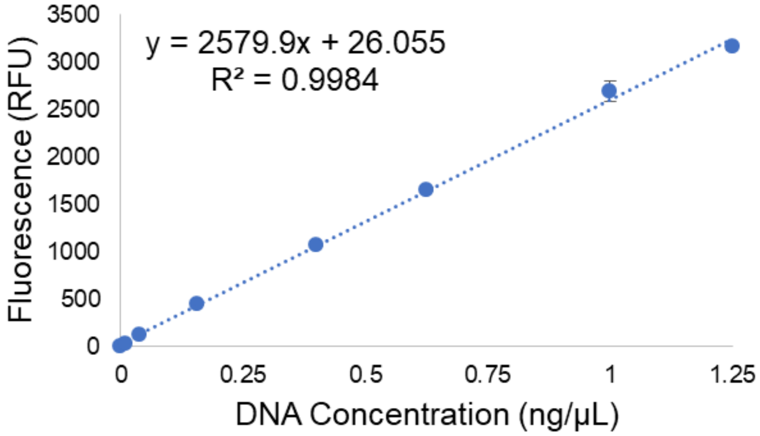


**S4 Fig.** The calibration curve for DNA quantification by the SYBR Green I intercalation fluorescence assay.
