# Supplementary material for: Vortex fluidics-mediated DNA rescue from formalin-fixed museum specimens
Source: PLoS One. 2020 Jan 30;15(1):e0225807. doi: 10.1371/journal.pone.0225807 (PMC6992170; doi:10.1371/journal.pone.0225807)
Supplement: S5 Fig — (PDF) [file pone.0225807.s005.pdf]

Part 5. Successful PCR's with variable gene target lengths and primers

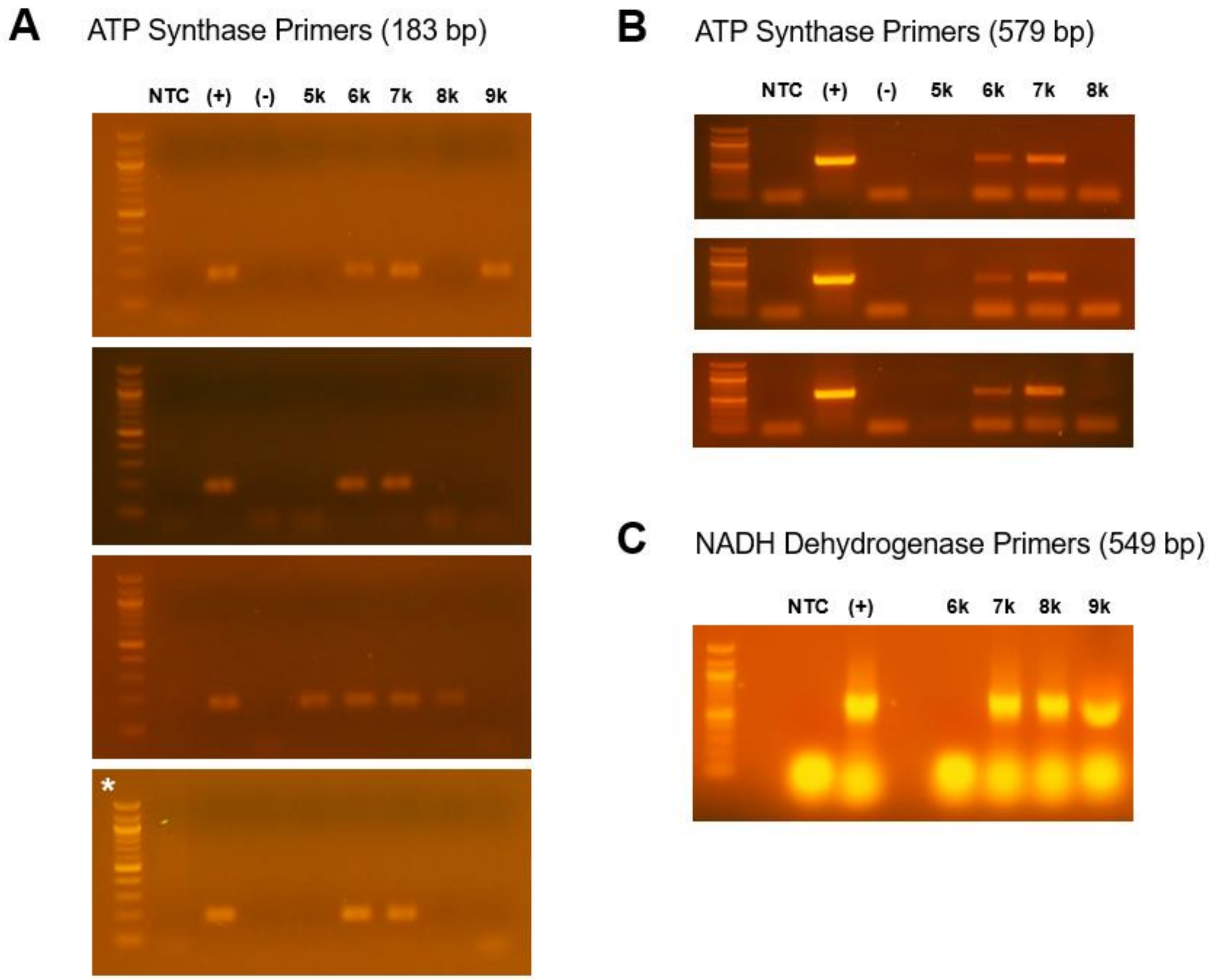

S5 Fig. qPCR and PCR (*****) of fDNA with **(A)** 183 bp ATP synthase amplicon primers showing multiple experiments to illustrate experimental consistency for the optimal VFD conditions reported here, but not 8 krpm rotational speeds, **(B)** 579 bp ATP synthase amplicon primers, and **(C)** 549 bp NADH dehydrogenase amplicon primers (forward: TCATCCATAGCACCAACCTTC; reverse: TGTTCAAGGCACTCTTATTTATATG; annealing temperature: 61 °C).
