# Supplementary material for: Vortex fluidics-mediated DNA rescue from formalin-fixed museum specimens
Source: PLoS One. 2020 Jan 30;15(1):e0225807. doi: 10.1371/journal.pone.0225807 (PMC6992170; doi:10.1371/journal.pone.0225807)
Supplement: S1 Table — (PDF) [file pone.0225807.s006.pdf]

S1 Table. Threshold cycle values (C_t) and endpoint fluorescence values of qPCR with the fDNA (Fig 2, S1 Fig, and S2 Fig).*

Sample	Reaction 1 C_t (fluorescence endpoint)	Reaction 2 C_t (fluorescence endpoint)	Reaction 3 C_t (fluorescence endpoint)
no template control	23.58 (270.38)	23.85 (243.87)	23.84 (255.38)
positive control	2.38 (1993.62)	2.57 (2095.95)	2.97 (1788.01)
negative control (non-VFD-processed)	23.85 (287.53)	23.87 (319.95)	24.26 (261.1)
5 krpm	N/A (-0.79)	N/A (0.35)	N/A (2.23)
6 krpm	31.20 (399.11)	33.68 (351.11)	31.34 (368.75)
7 krpm	29.81 (752.82)	29.87 (609.97)	29.12 (703.95)
8 krpm	N/A (321.42)	31.57 (328.76)	31.72 (332.93)

***** N/A = not applicable for samples that failed to amplify.
