# Supplementary material for: Vortex fluidics-mediated DNA rescue from formalin-fixed museum specimens
Source: PLoS One. 2020 Jan 30;15(1):e0225807. doi: 10.1371/journal.pone.0225807 (PMC6992170; doi:10.1371/journal.pone.0225807)
Supplement: S2 Table — (PDF) [file pone.0225807.s007.pdf]

Part 4. DNA quantification – qPCR and gel electrophoresis (Fig 4)

S2 Table. Threshold cycle values (C_t) and endpoint fluorescence values of qPCR with the fDNA (Fig 4).*

Sample	Reaction 1 C_t (fluorescence endpoint)	Reaction 2 C_t (fluorescence endpoint)	Reaction 3 C_t (fluorescence endpoint)
no template control	N/A (-0.05)	N/A (0.40)	37.30 (157.00)
positive control	20.80 (778.82)	22.07 (947.89)	22.84 (661.73)
negative control (non-VFD-processed)	N/A (1.38)	N/A (45.51)	N/A (-0.74)
5 krpm	N/A (-0.84)	N/A (23.88)	33.93 (656.01)
6 krpm	37.87 (542.35)	34.80 (987.87)	34.02 (603.64)
7 krpm	35.88 (732.61)	35.75 (925.38)	34.68 (589.66)
8 krpm	N/A (-0.37)	N/A (37.37)	35.15 (301.89)
9 krpm	39.13 (469.08)	N/A (0.06)	N/A (1.07)

***** N/A = not applicable for samples that failed to amplify.
